# Development, functional characterization and validation of methodology for GMP-compliant manufacture of phagocytic macrophages: A novel cellular therapeutic for liver cirrhosis

**DOI:** 10.1016/j.jcyt.2017.05.009

**Published:** 2017-09

**Authors:** Alasdair R. Fraser, Chloe Pass, Paul Burgoyne, Anne Atkinson, Laura Bailey, Audrey Laurie, Neil W.A. McGowan, Akib Hamid, Joanna K. Moore, Benjamin J. Dwyer, Marc L. Turner, Stuart J. Forbes, John D.M. Campbell

**Affiliations:** 1 Advanced Therapeutics, Scottish National Blood Transfusion Service, 21 Ellen's Glen Road, Edinburgh, United Kingdom; 2 Scottish National Blood Transfusion Service Cellular Therapy Development Centre, MRC Centre for Regenerative Medicine, The University of Edinburgh bioQuarter, Edinburgh, United Kingdom; 3 Red Cell Integrated Laboratory, Scottish National Blood Transfusion Service, Royal Infirmary of Edinburgh, Edinburgh, United Kingdom; 4 MRC Centre for Regenerative Medicine, University of Edinburgh, Edinburgh, United Kingdom

**Keywords:** cell therapy, cirrhosis, GMP, macrophage, process validation

## Abstract

**Background aims:**

Autologous macrophage therapy represents a potentially significant therapeutic advance for the treatment of severe progressive liver cirrhosis. Administration of macrophages has been shown to reduce inflammation and drive fibrotic scar breakdown and tissue repair in relevant models. This therapeutic approach is being assessed for safety and feasibility in a first-in-human trial (MAcrophages Therapy for liver CirrHosis [MATCH] trial).

**Methods:**

We outline the development and validation phases of GMP production. This includes use of the CliniMACS Prodigy cell sorting system to isolate CD14^+^ cells; optimizing macrophage culture conditions, assessing cellular identity, product purity, functional capability and determining the stability of the final cell product.

**Results:**

The GMP-compliant macrophage products have a high level of purity and viability, and have a consistent phenotypic profile, expressing high levels of mature macrophage markers 25F9 and CD206 and low levels of CCR2. The macrophages demonstrate effective phagocytic capacity, are constitutively oriented to an anti-inflammatory profile and remain responsive to cytokine and TLR stimulation. The process validation shows that the cell product in excipient is remarkably robust, consistently passing the viability and phenotypic release criteria up to 48 hours after harvest.

**Conclusions:**

This is the first report of validation of a large-scale, fully Good Manufacturing Practice–compliant, autologous macrophage cell therapy product for the potential treatment of cirrhosis. Phenotypic and functional assays confirm that these cells remain functionally viable for up to 48 h, allowing significant flexibility in administration to patients.

## Introduction

Liver cirrhosis is a major health problem in the Western world and a leading cause of mortality in the United Kingdom [1]. The disease is associated with a high level of morbidity due to the progressive tissue damage, fibrotic scarring and loss of liver function, and the only curative option for end-stage disease is liver transplantation. However, donor organ availability cannot meet demand, and often patients with end-stage liver disease are not eligible for transplantation. Those who do receive transplantation require lifelong immunosuppression with the increased health risks involved. Alternative therapies that prevent or delay the transition to terminal decompensated stages are urgently required [2].

The pathology of liver cirrhosis can be driven by numerous causative agents, including high alcohol consumption, obesity, metabolic disorders, viral infections or autoimmune disease, resulting in the progressive loss of healthy hepatocyte tissue and liver architecture, replaced by myofibroblast-derived fibrotic scarring [3], [4]. It has been increasingly recognized that if the agents driving liver damage are removed (e.g., alcohol, viruses), then liver fibrosis can be at least partially reversible, enabling liver regeneration to occur [5]. Animal models of liver regeneration after experimental liver damage have shown that macrophages play a key role in the control and repair of fibrotic liver disease [6]. The macrophage compartment of the liver is complex and dynamic and can be significantly modulated by disease [7]. Resident Kuppfer cells and recruited hematopoietic-derived macrophages have distinct but overlapping functions; however, it is clear that liver repair can be therapeutically reproduced by administration of monocyte-derived macrophages but not undifferentiated bone marrow cells or monocytes [8]. Indeed, inflammatory monocytes can contribute significantly to pathology [9]. Macrophage therapy can resolve carbon tetrachloride (CCL_4_)-mediated liver damage via decrease in myofibroblast levels and increased anti-inflammatory cytokine production [8]. Furthermore, macrophage-mediated matrix metalloprotease (MMP) release and phagocytosis is essential for fibrotic scar resolution [10]. Macrophages are also able to stimulate hepatic progenitor cells to proliferate and differentiate and replenish lost hepatocytes through Wnt and TWEAK signalling [11], [12]. Adoptive macrophage therapy therefore offers a significant potential treatment strategy for patients with cirrhotic liver disease to promote resolution of fibrosis and stimulate resolution.

A number of studies have outlined GMP-compliant protocols for the generation of adoptive cell therapies using leukocytes including dendritic cells [13], [14], [15] natural killer cells [16] or cytotoxic T cells [17], [18] *inter alia*. These studies largely harness the immune function of these cells. To date, few studies or trials have used macrophages for clinical cell therapy, focusing on either lung cancer [19] or bladder cancer [20], [21]. One study of acute spinal cord injury involved use of autologous blood-derived macrophages [22], but this product was small scale (<2 million cells).

We have recently demonstrated that CD14^+^ monocytes, collected by leukapheresis from cirrhotic donors, can be manufactured in a scalable manner into pro-resolution phenotype macrophages [23]. We are currently undertaking a first-in-human phase 0/1 safety and feasibility study to generate autologous CD14^+^ cell–derived macrophages under Good Manufacturing Practice (GMP) for re-infusion into cirrhotic patients (MAcrophage Therapy for liver CirrHosis [MATCH] trial) [24].

The MATCH trial requires the development of a consistent, well-characterized, autologous macrophage cell product in doses of multiples of 10^8^ cells. Here, we outline the development and validation for manufacturing of the MATCH product, including testing of GMP-grade media and growth factors for efficacy, analysis of cellular identity by multi-parameter flow cytometry, quantitative assessment of monocyte selection and macrophage purity and determination of a panel of markers which form the Release Criteria for the cell product. In addition, we examined the effects of monocyte cryopreservation on deriving macrophages for therapeutic use. Functional assays were conducted to quantify the phagocytic capacity of macrophages and their capacity for further polarization. This represents the first report of large-scale GMP-compliant macrophage manufacture and validation for first-in-human cell therapy of advanced liver cirrhosis.

## Methods

### Ethics and governance

Donor buffy coats as a source of healthy donor monocytes were provided by Scottish National Blood Transfusion Service (SNBTS) Blood Donor Centre, Edinburgh, United Kingdom, under SNBTS Sample Governance 13-12 and 14-02. For full-scale GMP process optimization and validation, peripheral blood mononuclear cells (PBMCs)were collected by leukapheresis in the SNBTS Clinical Apheresis Unit, Royal Infirmary of Edinburgh. Ethical approval was granted from the South East Scotland Research Ethics Committee 02. Informed consent for apheresis donation was obtained in accordance with the Helsinki Declaration.

### Cell preparation

CD14 selection from PBMCs: PBMCs were separated from normal donor buffy coats by density centrifugation using Histopaque 1077 (Sigma). After washing, CD14^+^ monocytes were isolated from the mononuclear cell fraction using CliniMACS GMP-grade CD14 microbeads and LS separation magnetic columns (Miltenyi Biotec). Briefly, cells were re-suspended to appropriate concentration in PEA buffer (phosphate-buffered saline [PBS] plus 2.5 mmol/L ethylenediaminetetraacetic acid [EDTA] and human serum albumin [0.5% final volume of Alburex 20%, Octopharma]), incubated with CliniMACS CD14 beads per manufacturer's instructions, then washed and passed through a magnetized LS column. After washing, the purified monocytes were eluted from the demagnetized column, washed and re-suspended in relevant medium for culture.

Isolation of CD14^+^ cells from leukapheresis: PBMCs were collected by leukapheresis from cirrhotic donors who gave informed consent to participate in the study. Eligibility criteria were age range of 18 to 75 years, and cirrhosis was defined by any one of the following: previous liver biopsy confirming histological features of cirrhosis, transient elastography (Fibroscan) > 18 kPa and/or clinical and radiological features that in the opinion of the clinical lead correlated with a diagnosis of cirrhosis. Exclusion criteria were viral hepatitis, average alcohol ingestion >21 units/week (male) or >14 units/week (female), ascites not well controlled with diuretic therapy in the preceding 3 months, encephalopathy requiring hospitalisation for treatment in the previous 3 months, portal hypertensive bleeding in the preceding 3 months, hepatocellular carcinoma, other cancer within the previous 5 years, previous liver transplant or currently on the waiting list, the presence of a clinically relevant acute illness that might compromise safe presentation and pregnancy and/or breast-feeding.

Leukapheresis of peripheral blood for mononuclear cells (MNCs) was carried out using an Optia apheresis system by sterile collection. A standard collection program for MNC was used, processing 2.5 blood volumes.

Isolation of CD14 cells was carried out using a GMP-compliant functionally closed system (CliniMACS Prodigy system, Miltenyi Biotec). Briefly, the leukapheresis product was sampled for cell count and an aliquot taken for pre-separation flow cytometry. The percentage of monocytes (CD14^+^) and absolute cell number were determined, and, if required, the volume was adjusted to meet the required criteria for selection (≤20 × 10^9^ total white blood cells; <400 × 10^6^ white blood cells/mL; ≤3.5 × 10^9^ CD14 cells, volume 50–300 mL). CD14 cell isolation and separation was carried out using the CliniMACS Prodigy with CliniMACS CD14 microbeads (medical device class III), TS510 tubing set and LP-14 program. At the end of the process, the selected CD14^+^ positive monocytes were washed in PBS/EDTA buffer (CliniMACS buffer, Miltenyi) containing pharmaceutical grade 0.5% human albumin (Alburex), then re-suspended in TexMACS (or comparator) medium for culture.

### Cell count and purity

Cell counts of total MNCs and isolated monocyte fractions were performed using a Sysmex XP-300 automated analyzer (Sysmex). Assessment of macrophage numbers was carried out by flow cytometry with TruCount tubes (Becton Dickinson) to determine absolute cell number, as the Sysmex consistently underestimated the number of macrophages. The purity of the separation was assessed using flow cytometry (FACSCanto II, BD Biosciences) with a panel of antibodies against human leukocytes (CD45-VioBlue, CD15-FITC, CD14-PE, CD16-APC), and product quality was assessed by determining the amount of neutrophil contamination (CD45int, CD15pos).

### Cell culture—development of cultures with healthy donor samples

Optimal culture medium for macrophage differentiation was investigated, and three candidates were tested using buffy coat monocytes; Dulbecco's Modified Eagle's Medium (DMEM; Life Technologies) supplemented with 5% AB serum (SNBTS), plus two chemically defined serum-free culture media, AIM-V (Thermo Fisher) and TexMACS (Miltenyi). The experiments were carried out with buffy coat monocytes cultured in each medium for 7 days in a humidified atmosphere at 37°C and 5% CO_2_ with 100 ng/mL premium grade Macrophage Colony Stimulating Factor (M-CSF, Miltenyi). Cells were seeded at a density of 1.8 × 10^7^ cells/well in 4 mL medium in six-well tissue culture plates and re-fed at days 3–4. The best response was seen with TexMACS and this was used for all subsequent experiments (see Results section). A GMP-compliant M-CSF from R&D Systems was tested at 100 ng/mL compared with the research grade M-CSF used in the previous study [23] and was found to be comparable to the premium grade M-CSF previously used (see Results). The GMP-grade M-CSF was subsequently used in all further experiments with buffy coats. A comparison was also made of deriving macrophages from fresh and frozen/thawed monocytes (see Results section). Monocytes were isolated from buffy coats, then an aliquot was frozen in cryopreservation medium (Cryostor CS10, Sigma) and the remainder was cultured fresh. The frozen aliquot was thawed several days later and cultured as before. The macrophage yield and surface phenotype was assessed by counting and flow cytometry (see “Flow cytometry characterization” section).

### Full-scale process validation with patient samples

Monocytes cultured from leukapheresis from Prodigy isolation were cultured at 2 × 10^6^ monocytes per cm^2^ and per mL in culture bags (MACS GMP differentiation bags, Miltenyi) with GMP-grade TexMACS (Miltenyi) and 100 ng/mL M-CSF. Monocytes were cultured with 100 ng/mL GMP-compliant recombinant human M-CSF (R&D Systems). Cells were cultured in a humidified atmosphere at 37°C, with 5% CO_2_ for 7 days. A 50% volume media replenishment was carried out twice during culture (days 2 and 4) with 50% of the culture medium removed, then fed with fresh medium supplemented with 200 ng/mL M-CSF (to restore a final concentration of 100 ng/mL).

### Cell harvesting

For normal donor-derived macrophages, cells were removed from the wells at day 7 using Cell Dissociation Buffer (Gibco, Thermo Fisher) and a pastette. Cells were resuspended in PEA buffer and counted, then approximately 10^6^ cells per test were stained for flow cytometry.

Leukapheresis-derived macrophages were removed from the culture bags at day 7 using PBS/EDTA buffer (CliniMACS buffer, Miltenyi) containing pharmaceutical grade 0.5% human albumin from serum (HAS; Alburex). Harvested cells were resuspended in excipient composed of two licensed products: 0.9% saline for infusion (Baxter) with 0.5% human albumin (Alburex).

### Flow cytometry characterization

Monocyte and macrophage cell surface marker expression was analyzed using either a FACSCanto II (BD Biosciences) or MACSQuant 10 (Miltenyi) flow cytometer. Approximately 20 000 events were acquired for each sample. Cell surface expression of leukocyte markers in freshly isolated and day 7 matured cells was carried out by incubating cells with specific antibodies (final dilution 1:100). Cells were incubated for 5 min with FcR block (Miltenyi) then incubated at 4°C for 20 min with antibody cocktails. Cells were washed in PEA, and dead cell exclusion dye DRAQ7 (BioLegend) was added at 1:100.

Cells were stained for a range of surface markers as follows: CD45-VioBlue, CD14-PE or CD14-PerCP-Vio700, CD163-FITC, CD169-PE and CD16-APC (all Miltenyi), CCR2-BV421, CD206-FITC, CXCR4-PE and CD115-APC (all BioLegend), and 25F9-APC and CD115-APC (eBioscience). Both monocytes and macrophages were gated to exclude debris, doublets and dead cells using forward and side scatter and DRAQ7 dead cell discriminator (BioLegend) and analyzed using FlowJo software (Tree Star).

From the initial detailed phenotyping, a panel was developed as Release Criteria (CD45-VB/CD206-FITC/CD14-PE/25F9-APC/DRAQ7) that defined the development of a functional macrophage from monocytes. Macrophages were determined as having mean fluorescence intensity (MFI) five times higher than the level on day 0 monocytes for both 25F9 and CD206. A second panel was developed which assessed other markers as part of an Extended Panel, composed of CCR2-BV421/CD163-FITC/CD169-PE/CD14-PerCP-Vio700/CD16-APC/DRAQ7), but was not used as part of the Release Criteria for the cell product.

### Functional characterization

Both monocytes and macrophages from buffy coat CD14 cells were tested for phagocytic uptake using pHRodo beads, which fluoresce only when taken into acidic endosomes. Briefly, monocytes or macrophages were cultured with 1–2 uL of pHRodo *Escherichia coli* bioparticles (Life Technologies, Thermo Fisher) for 1 h, then the medium was taken off and cells washed to remove non-phagocytosed particles. Phagocytosis was assessed using an EVOS microscope (Thermo Fisher), images captured and cellular uptake of beads quantified using ImageJ software (NIH freeware, https://imagej.nih.gov/ij*).*

The capacity to polarize toward defined differentiated macrophages was examined by treating day 7 macrophages with interferon (IFN)-γ (50 ng/mL) or interleukin (IL)-4 (20 ng/mL) for 48 h to induce polarization to M1 or M2 phenotype (or M[IFNγ] versus M[IL-4], respectively). After 48 h, the cells were visualized by EVOS bright-field microscopy, then harvested and phenotyped as before.

Further analysis was performed on the cytokine and growth factor secretion profile of macrophages after generation and in response to inflammatory stimuli. Macrophages were generated from healthy donor buffy coats as before, and either left untreated or stimulated with tumor necrosis factor (TNF)-α (50 ng/mL, Peprotech) and polyinosinic:polycytidylic acid (poly I:C, a viral homolog which binds TLR3, 1 µg/mL, Sigma) to mimic the conditions present in the inflamed liver, or lipopolysaccharide (LPS, 100 ng/mL, Sigma) plus IFN-γ (50 IU/mL, Peprotech) to produce a maximal macrophage activation. Day 7 macrophages were incubated overnight and supernatants collected and spun down to remove debris, then stored at −80°C until testing. Secretome analysis was performed using a 27-plex human cytokine kit and a 9-plex matrix metalloprotease kit run on a Magpix multiplex enzyme-linked immunoassay plate reader (BioRad).

### Product stability

Various excipients were tested during process development including PBS/EDTA buffer; PBS/EDTA buffer with 0.5% HAS (Alburex), 0.9% saline alone or saline with 0.5% HAS. The 0.9% saline (Baxter) with 0.5% HAS excipient was found to maintain optimal cell viability and phenotype (data not shown). The stability of the macrophages from cirrhotic donors after harvest was investigated in three process optimization runs, and a more limited range of time points assessed in the process validation runs (n = 3). After harvest and re-suspension in excipient (0.9% saline for infusion, 0.5% human serum albumin), the bags were stored at ambient temperature (21–22°C) and samples taken at 0, 2, 4, 6, 8, 12, 24, 30 and 48 h post-harvest. The release criteria antibody panel was run on each sample, and viability and mean fold change from day 0 was measured from geometric MFI of 25F9 and CD206.

### Statistical analysis

Results are expressed as mean ± SD. The statistical significance of differences was assessed where possible with the unpaired two-tailed *t*-test using GraphPad Prism 6. Results were considered statistically significant when the *P* value was <0.05.

## Results

In this study, we have defined a number of aspects of the cultured macrophage, modeling first with healthy donor samples to carry out extensive preclinical characterization of normal macrophages. This knowledge has then been applied to full-scale leukapheresis collections from patients with liver cirrhosis to optimize and validate the final cell therapy manufacturing procedure.

### Optimizing GMP-compliant culture of monocytes

**Figure 1:**
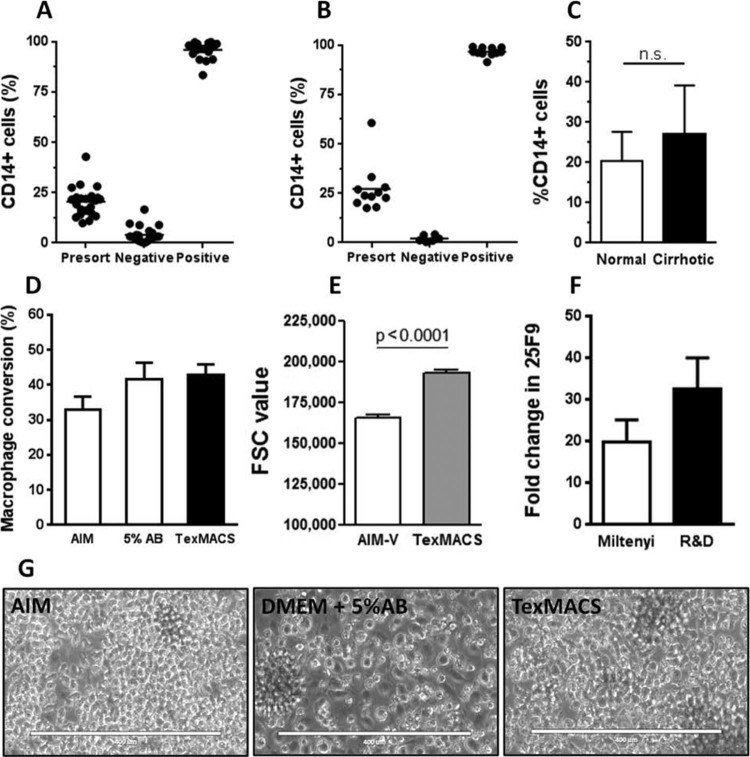
Initial optimization of GMP macrophage culture from normal donors. (A) CliniMACS CD14 bead isolation of monocytes using LS columns generated highly purified CD14 fractions (n = 26). (B) CliniMACS Prodigy CD14 selection produced equally highly enriched CD14^+^ fractions from apheresis collection from cirrhotic volunteers. (C) Cirrhotic patients showed a trend to higher CD14 numbers in peripheral blood (n = 11, *P* = 0.108). (D) Although not significant, TexMACS produced the highest and most consistent yield of macrophages (n = 6–10). (E) Macrophages from TexMACS culture were also significantly larger in size than those cultured in AIM-V medium (*P *<* *0.0001, n = 5). (F) The GMP-grade M-CSF from research and development generated a similar yield of macrophages to standard M-CSF but with stronger expression of macrophage marker 25F9 (n = 6). (G) Microscopic imaging indicates that DMEM + 5% AB produced clumping and sticking in macrophage cultures, unlike those generated in defined media.

The sorting efficiency using CD14 CliniMACS beads was extremely high, and results were comparable across all methods, whether the cells were sourced from buffy coat or leukapheresis from cirrhotic patients; bead-labeled cells were manually labeled and isolated over LS columns or processed using the completely automated CliniMACS prodigy system. Flow cytometric analysis of the pre-sort and positive and negative fractions indicated that from an initial mean of 20.5% monocytes, the positive fraction was highly enriched (95.9%), with minimal loss in the negative fraction (4.1%) from 26 normal donor isolates using CliniMACS CD14 reagents and LS columns (Figure 1A). Very similar figures were seen in cirrhotic donor samples separated using the CliniMACS Prodigy (Figure 1B), although cirrhotic donors had a higher (although not significant) initial mean percentage of monocytes in their collections (Figure 1C). We have previously reported the manufacture of macrophages in AIM-V complete culture medium. However, the antibiotic content of this medium was not compatible with the testing of product sterility by assessment of bacterial growth and therefore we evaluated the antibiotic-free TexMACS complete medium as a comparator. GMP-grade TexMACS culture medium demonstrated a consistently higher conversion rate to macrophages compared with AIM-V, although this did not reach statistical significance (Figure 1D). Culture in DMEM plus 5% AB serum also resulted in a comparable rate of macrophage conversion; however, DMEM cultured macrophages were also strongly adherent to the substrate, which resulted in cell loss during harvesting (Figure 1G). In addition, monocytes cultured in TexMACS produced significantly larger macrophages as calculated using forward scatter from flow cytometry (Figure 1E). Initial development experiments used a premium research grade M-CSF (Miltenyi) to supplement the culture medium. This was compared with a GMP-grade M-CSF (R&D Systems), both used at 100 ng/mL. There were no significant differences in morphology or macrophage yield when culturing with either source, but higher expression of the macrophage marker 25F9 was seen in macrophages generated with GMP-grade M-CSF (Figure 1F). Therefore, we considered TexMACS medium (Miltenyi) supplemented with GMP-grade M-CSF (R&D Systems) to be the optimal medium for the production of GMP grade macrophages.

### Derivation of macrophages from frozen monocytes

**Figure 2:**
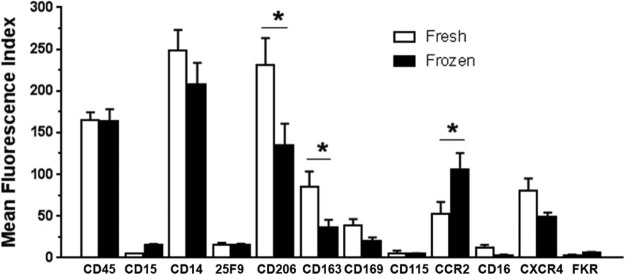
Phenotyping of fresh versus frozen monocytes for GMP macrophages. Buffy coat monocytes were isolated, an aliquot frozen and stored in LN_2_ and the remainder used to generate macrophages. After thawing, monocytes were cultured to macrophages and phenotype compared. Although many markers are unchanged in macrophages made from thawed monocytes, CD206 and CD163 are significantly reduced and CCR2 significantly increased in macrophages from frozen monocytes (n = 10).

For therapeutic use, freezing aliquots of monocytes to generate multiple macrophage doses would be attractive. We assessed whether there were any significant differences in macrophage yield or phenotype that would preclude the use of frozen stocks. Our data indicated that there were differences in both yield and in phenotype. In particular, there were significant drops in the expression of CD206 and CD163 (Figure 2). There was also a converse increase in CCR2 expression (Figure 2). This would suggest that the macrophages from frozen monocytes develop a more M1-like, classically activated phenotype. Additionally, the mean viability of macrophages from thawed monocytes was significantly lower than from fresh monocytes in these experiments (viability 45.5% versus 75.2% in fresh macrophages, n = 4–6, *P* = 0.0098). It was concluded that the poor viability (and therefore yields) and less advantageous phenotype precluded the further development of the macrophage product from frozen cells for initial clinical use.

### Extended functional analysis: healthy donor macrophages

**Figure 3:**
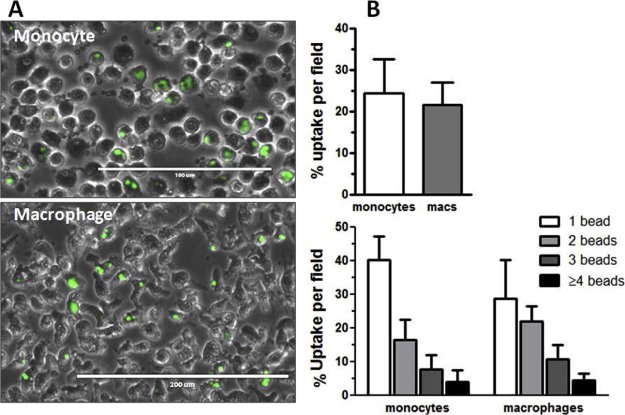
Phagocytosis of monocytes and macrophages. (A) EVOS image of bright-field/fluorescein isothiocyanate fluorescence indicating pHRodo bead uptake (magnification ×100). (B) Quantification of uptake indicates that there is no significant difference in uptake of beads by monocytes or macrophages, and no difference in number of beads that were taken up (n = 4). Data are expressed as mean ± SD.

Functional characterization of normal macrophages investigated the capacity of the cells to take up particles. Using pHRodo beads in these studies gives an accurate assessment because the beads are clear until phagocytosed and fluoresce once exposed to the acidic environment of the phagolysosome. Phagocytosis was quantified from EVOS images (Figure 3A), assessing total cell numbers per field, total cells containing fluorescent beads, and then number of beads per cell. Quantification of phagocytosis using this method demonstrated that there was no difference in uptake between monocytes and macrophages (Figure 3B). Further analysis indicated that stimulation of macrophages with TNF-α and poly I:C had no significant effect on phagocytosis (data not shown).

**Figure 4:**
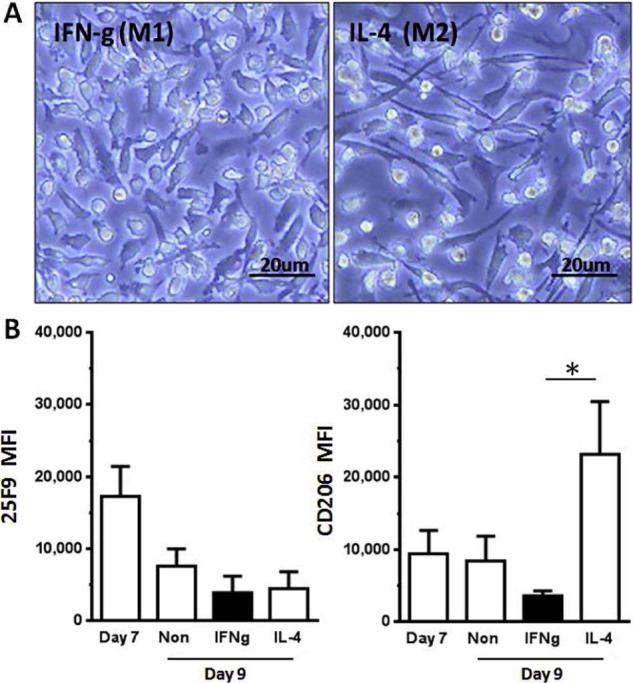
Microscopic and phenotypic changes in polarized macrophages. (A) EVOS bright-field image of polarized macrophages shows distinct differences in morphology, with angular, spiky M1/M(IFNγ) and elongated smooth spindle-shaped M2/M(IL-4) macrophages (magnification ×400). (B) Flow cytometric analysis shows no significant difference in macrophage marker 25F9 in all, but IL-4 stimulated macrophages significantly increase their CD206 expression, but decreased in IFN-γ treated macrophages (n = 5, mean ± SD, *P *<* *0.05).

Our phenotype analysis indicated that the macrophages at harvest are not polarized, but they do demonstrate high CD206 and CD169 expression characteristic of alternatively activated macrophages (Figure 6). We used IFN-γ and IL-4 stimulation to determine whether the macrophages at harvest were capable of responding to polarizing cytokine exposure. At day 2 of polarization, there was a morphological difference in the cultures, with IFN-γ–treated macrophages showing rounded, angular shape (Figure 4A), whereas IL-4–treated macrophages showed characteristic spindle-shaped morphology [25]. Although there was no significant difference in the 25F9 marker expression between the cultures, the IL-4–treated cells were able to significantly up-regulate CD206, characteristic of polarized M2a macrophages (Figure 4B). This polarization is characteristic of macrophages *in vitro* and does not necessarily predict functional capacity in recipients.

**Figure 5:**
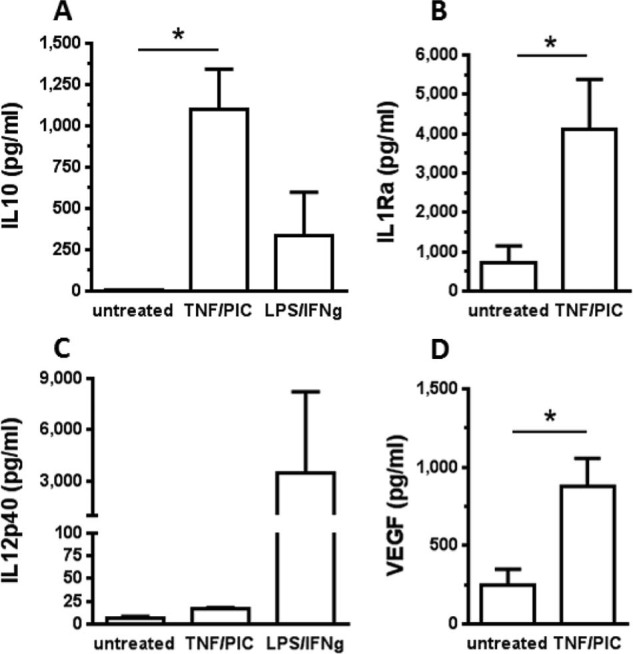
Secretome analysis of healthy donor macrophages. Day 7 macrophages (n = 3) were untreated or stimulated for 12 h with TNF-α and poly I:C (PIC) or IFN-γ and LPS, then analyzed by BioRad multiplex enzyme-linked immunoassay. Both IL-10 (A) and IL-1Ra (B) were significantly up-regulated by TNF-α/PIC, as was VEGF (D). The IL-12p40 level (C) was not affected by TNF-α/PIC but was significantly increased by LPS/IFN-g. Data are expressed as mean + SD, *P <* *0.05.

A final aspect of cell product assessment was determination of macrophage factor secretion profile at rest and after stimulation (Figure 5). Macrophages expressed low levels of IL-1Ra, IL-10 and vascular endothelial growth factor (VEGF) at rest. Stimulation with TNF-α and poly I:C was used to mimic an inflamed environment and led to significant increases in production of the pro-regenerative/anti-inflammatory factors VEGF, IL-10 and IL-1Ra, but no increase in IL-12. IL-10 expression was not increased strongly by LPS/IFN-γ stimulation, but conversely IL-12 expression was strongly increased by LPS/IFN-γ.

TNF-α/poly I:C stimulation increased expression of CCL3, 4 and 5 but with no significant increase in CCL2 (supplementary Figure S1). Macrophages constitutively expressed high levels of MMP 7, 9 and 12 with low expression of MMP3. This MMP expression was not modulated significantly after stimulation (supplementary Figure S2) or polarization, and there was negligible expression of MMPs 1, 7, 8, 10 and 11 (data not shown).

### Process validation: cirrhotic patients

**Figure 6:**
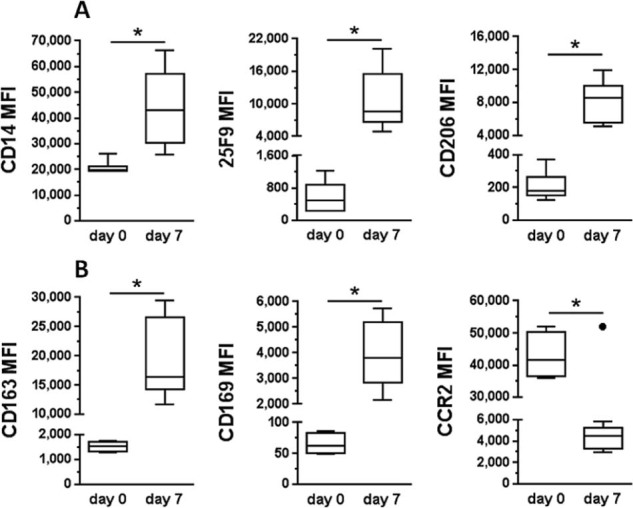
Phenotype panels for clinical grade macrophages taken from cirrhotic patients (n = 7). (A) Release criteria showing MFI increase between day 0 monocytes and day 7 macrophages. (B) Extended panel including two further scavenger receptors (CD163/CD169) plus a marker for macrophage migration. Note that a single outlier on the CCR2 day 7 data was excluded from analysis after Grubb's test (●). Data are presented as mean ± SD, *P *<* *0.05.

The cell culture and identity data were used to design the GMP process, which was then validated. A set of markers was chosen from the broad phenotyping panel to use as product Release Criteria inclusive of cell identity and functional markers. These were expression of CD45 and CD14 for lineage determination and 25F9 as a marker of macrophage maturity. In addition, CD206 was chosen as a surrogate marker for phagocytosis and scavenging capacity, which would effectively identify the development of suitable functional macrophages. The viability stain DRAQ7 was included as a final component of the GMP Release Criteria panel. For further information on the cell product, we used a second phenotyping set, termed the Extended Panel, which assesses expression of CD163, CD169 and CCR2. This panel was validated on leukapheresis donations from seven cirrhotic volunteers. CD14^+^ cells were selected using the CliniMACS Prodigy device, and the cells were cultured as described earlier in the article. The levels of expression of each marker are shown on day 0 enriched monocytes and corresponding day 7 macrophages in Figure 6. Differentiated cells retain CD45^+^ CD14^+^ expression and 25F9, CD206, CD169 and CD163 was significantly elevated in macrophages. CCR2 becomes significantly down-regulated in macrophages compared with monocytes. We also assessed migratory capacity of the macrophages post-harvest using trans-well chemotaxis assay and confirmed that they retained the ability to migrate to suitable targets *in vitro* despite the down-regulation of CCR2 (data not shown).

**Figure 7:**
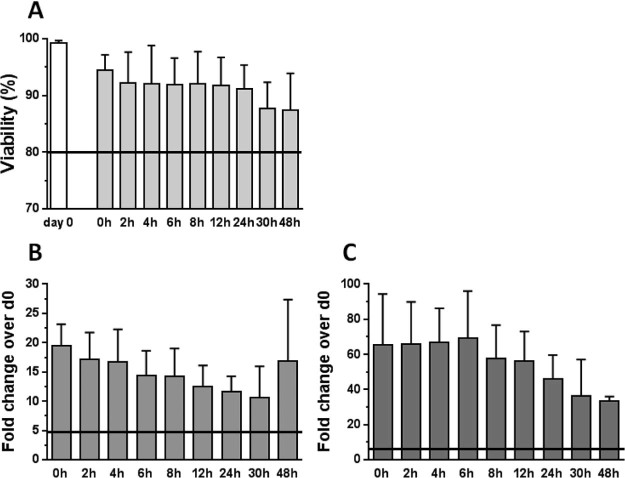
Stability of GMP cirrhotic patient macrophages. (A) Viability shows no significant decrease even after 48 h at ambient temperature. (B) Expression of 25F9 decreases steadily over time but even at 48 h remains well above the fivefold increase required for validity. (C) CD206 remains high until approximately 24–30 h after harvest then drops more rapidly, but also remains well above the fivefold threshold established for the trial criteria (n = 3).

Stability studies were performed on macrophages stored at controlled room temperature (21–22°C) in optimal excipient, as determined during process optimization (clinical grade 0.9% saline with 0.5% HAS). After sampling at each time point, the cells were assessed for phenotype and viability. Data from all three process optimization runs indicated that both cell viability and phenotype (25F9/CD206 MFI) were maintained to 48 h post-harvest (Figure 7), indicating that the process reproducibly produces a very stable cell product. Release Criteria were thus established that the macrophages should be CD45/CD14 positive, with a viability greater than 80%, and have a MFI for 25F9 and CD206 more than fivefold higher than the MFI of the original monocytes at day 0. All final results for Release Criteria process validation including stability are detailed in Table I.

**Table I:** Data from process validation runs 1–3.

Acceptance criteria	Expected result	Actual result		
		Patient 1	Patient 2	Patient 3
Selection (D0) CD14 yield	≥40%	41%	59%	64%
Final macrophage yield (viable cell number)	1 × 10^7^ to 1 × 10^9^	5.11 × 10^8^	5.73 × 10^8^	2.9 × 10^8^
Harvest (D7) Macrophage viability	≥80%	87%	95%	87%
Harvest (D7) 25F9 MFI fold increase	≥5	17	19	26
Harvest (D7) CD206 MFI fold increase	≥5	33	28	82
Stability (48 h) Macrophage Viability	≥80%	91%	88%	86%
Stability (48 h) 25F9 MFI fold increase	≥5	18	8	11
Stability (48 h) CD206 MFI fold increase	≥5	21	19	41

Details include the parameters that will be used as release criteria for final product.

## Discussion

Macrophages derived from CD14^+^ monocytes sourced from peripheral blood or leukapheresis can reverse experimental liver fibrosis [11], [23], [26]. In contrast, undifferentiated CD14^+^ monocytes can exacerbate liver disease, as shown by CCR2-mediated blockade of monocyte recruitment in models of liver disease [27]. In our previous study, we showed that equivalent cells can be generated from healthy blood donors and cirrhotic patients [23]. In this study, we show that macrophages can be generated in xeno-free, fully GMP-compliant conditions at a scale of multiples of 10^8^ cells and rigorously validated for use in a first-in-human clinical trial. Throughout the current study, we have determined the criteria of reproducibility and function of the macrophages using healthy donor cells and then applied these to cells from cirrhotic volunteer donors to establish and validate the manufacturing procedure for clinical use.

In this study and our previous feasibility study [23], we have used CD14 microbead selection to isolate monocytes as a manufacturing raw material because this technique has been shown to give high yields of pure monocytes, suitable for further manufacturing [14]. CD14 selection has no appreciable adverse effect on function such as response to TLR ligation or CD40 ligation [28] and yielded cells efficient at reversing cirrhosis in experimental models [23]. The CliniMACS Plus device has been widely used for isolation for CD14^+^ monocytes, principally for the preparation of monocyte-derived dendritic cells [13], [29]. Although effective, this methodology involves a series of manual washes and incubations, which can reduce overall cell yield. In this study, we report the first use of the CliniMACS Prodigy device to automate large parts of the CD14^+^ isolation process, with only a single final wash of harvested cells to remove buffer before cell culture. We were easily able to replicate our translation yields of monocytes using small-scale selection over MACS LS columns to the CliniMACS Prodigy, with identically high levels of CD14^+^ enrichment using both methods. The automated procedure shortens processing time, reduces the amount of manual handling and led to average yields of many multiples of 10^8^ CD14^+^ cells. This therefore makes it feasible to manufacture macrophage products at significant scale, with fewer manual handling steps. It is worth noting that cirrhotic donors have high numbers of circulating monocytes, commonly more than 30% of the Total Nucleated Cells, which is considerably more than reported values for healthy donors or the patients donating for CD14 processing [13], [29]. Very high starting numbers of target cells can potentially reduce overall selection efficiency due to overwhelming the capacity of reagent and selection columns. Despite the high monocyte numbers in cirrhotic patients, the CliniMACS Prodigy system was able to reproducibly isolate sufficient cells to meet our manufacturing criteria of a yield of at least 40% CD14^+^ cells.

We have previously reported that this process could be carried out using GMP-compatible reagents including human donor serum and carrier-free cytokines. Here we have further adapted the process to eradicate serum entirely and used only growth factor–supplemented GMP-standard defined culture medium with identical yield to serum-supplemented medium. In addition to the anticipated increased manufacturing reproducibility of using defined medium, we found that harvesting macrophages was more efficient with the eradication of donor serum from the cell culture because the final product is more loosely adherent to the bag. This is of considerable benefit in minimizing cell loss during harvest of CD14-derived cell products. With the increasing availability of GMP-grade cytokines, we were also able to validate our method using GMP-grade M-CSF with no detriment to final cell yield or phenotype. The higher mean fluorescence levels of the macrophage marker 25F9 on cells cultured in GMP-grade cytokine were of practical benefit in determining fold-change over baseline as a release criterion by flow cytometry.

We have validated flow cytometry as the principal functional Release Criteria for the macrophage product. This was used as a single platform to analyze the product pre-release, including absolute cell counting (TruCount) and viability (exclusion of DRAQ7). In a product such as this, where differentiation from a precursor to a final phenotype is the aim of the manufacturing process, the use of fold change of the whole population MFI was felt to be more relevant than use of arbitrary percentage positivity on the basis of quadrant gating. We were able to generate robust limits for the increase in 25F9 to define the macrophage identity, plus expression of the phagocytosis-associated marker CD206 as the minimum release criteria. Of note, it was not possible to establish a bead phagocytosis assay to define a macrophage phenotype that differed from undifferentiated monocytes because both performed identically in this assay. The use of acid phagosome–activated fluorescent beads increased the accuracy of this assay because only internalized beads, and not surface-bound beads, were quantified. However, CD206, CD169 and CD163 expression was definitively confined to macrophages and the former adopted as a surrogate marker of phagocytic function for product release in the trial.

In the MATCH trial, provided that dose escalation is successfully completed, the main treatment arm will consist of three infusions on a monthly basis of the maximum tolerated dose of macrophages. This treatment regime will consist of multiples of 10^8^ macrophages per dose. For convenience, this would ideally have been delivered through a single leukapheresis collection, followed by selection and cryopreservation of CD14^+^ cells, then manufacture of three products from thawed monocytes. However, the data we generated in this study did not support this manufacturing strategy because macrophage yield per input CD14^+^ cells was only about 40%, which is insufficient to generate multiple doses of up to10^9^ macrophages. The phenotype of the macrophages after differentiation from cryopreserved CD14 cells was also altered with significant drops in the expression of CD206 and CD163 compared with fresh product. There was also a converse increase in CCR2 expression, a chemokine receptor associated with trafficking to inflamed sites, highly expressed on steady state and inflammatory monocytes and associated potential exacerbation of disease [7]. All of these factors meant that thawed CD14-derived macrophages did not meet our manufacturing criteria. It is likely that specialist cryopreservation strategies will be needed to support better yield and function of thawed monocytes [30].

Having defined our methods and assays for the macrophage product, we were able to use this to perform extended analysis on the macrophages, validate the full-scale manufacturing process and establish product stability. Our extended analysis indicated that macrophages generated by this xeno-free process had both a pro-regenerative phenotype and were migration competent. The cells stably expressed a variety of MMP and CC chemokines, known to be associated with the *in vivo* regenerative function of macrophages in liver fibrosis [5], [11], [23]. Increases in the production of anti-inflammatory cytokines IL-10 and IL-1Ra and the pro-angiogenic factor VEGF, but not IL-12 from the macrophages in response to TNF-driven inflammation signals, also confirmed the anti-inflammatory phenotype [31]. The cells retained a degree of plasticity, with their expression of the scavenger molecule CD206 considerably up-regulated by incubation with IL-4 but slightly decreased by incubation with IFN-γ. This polarization competence was demonstrated on cultured macrophages but has not been confirmed *in vivo*. However, phenotype and cytokine profiles in response to inflammatory factors present in inflamed liver leads us to conclude that the transfused macrophages will contribute to damage resolution rather than exacerbate pathology.

Final manufacturing process validation runs were successfully carried out, and we were able to manufacture a minimum of 2.9 × 10^8^ and maximum of 5.7 × 10^8^ macrophages from cirrhotic volunteers. Moreover, the product proved to be extremely stable for up to 48 h in excipient at controlled room temperature. This stability will allow significant flexibility in scheduling infusion of the macrophages to patients and could be used to underpin the use of the product in multiple centers. These validation and stability data were used to prepare the relevant validation documentation for submission to the Medicines and Healthcare Products Regulatory Agency as part of the Investigational Medicinal Product Dossier and a Clinical Trial Authorisation was subsequently issued for the MATCH trial.
