# Supplementary material for: Development, functional characterization and validation of methodology for GMP-compliant manufacture of phagocytic macrophages: A novel cellular therapeutic for liver cirrhosis
Source: Cytotherapy. 2017 Sep;19(9):1113–24. doi: 10.1016/j.jcyt.2017.05.009 (PMC5571439; doi:10.1016/j.jcyt.2017.05.009)
Supplement: Appendix S1 — Figures S1 and S2. [file mmc1.docx]

Supplementary Figure 1. Chemokine expression by healthy donor untreated macrophages (U) and after stimulation with TNF-α and poly (I:C) (T/P). Although CCL3, 4 and 5 show significant up-regulation after stimulation, there was no modulation of CCL2 expression. Data are expressed as mean ± SD (n = 3, *P* < 0.05)

Supplementary Figure 2. Matrix metalloprotease (MMP) expression by healthy donor untreated macrophages (U) and after stimulation overnight with TNF-α and poly (I:C) (T/P). No significant difference was seen in expression levels after stimulation. Data are expressed as mean + SD (n = 4, *P* < 0.05)
